# Spontaneous retroperitoneal hematoma treated with transarterial embolization: a systematic review and metanalysis

**DOI:** 10.1186/s42155-024-00462-6

**Published:** 2024-05-25

**Authors:** Francesco Tiralongo, Stefano Toscano, Cristina Mosconi, Roberto Iezzi, Francesco Giurazza, Davide Giuseppe Castiglione, Daniele Falsaperla, Francesco Vacirca, Corrado Ini’, Fabio Corvino, Salvatore Lavalle, Massimo Venturini, Pietro Valerio Foti, Stefano Palmucci, Antonio Basile

**Affiliations:** 1 Radiology Unit 1, Department of Medical Surgical Sciences and Advanced Technologies “GF Ingrassia”, University Hospital Policlinico “G. Rodolico-San Marco”, University of Catania, Catania, 95123 Italy; 2 Department of Radiology, IRCCS Azienda Ospedaliero Universitaria Di Bologna, via Albertoni 15, Bologna, 40138 Italy; 3 Dipartimento di Diagnostica per Immagini, Radioterapia Oncologica ed Ematologia-Istituto di Radiologia, Fondazione Policlinico Universitario A. Gemelli IRCCS, l.go A gemelli 8, Rome, 00168 Italy; 4 Istituto di Radiodiagnostica, Università Cattolica del Sacro Cuore, Rome, 00168 Italy; 5 Interventional Radiology Department, Cardarelli Hospital of Naples, Naples, 80131 Italy; 6 Faculty of Medicine and Surgery, University of Enna Kore, Enna, 94100 Italy; 7 Department of Diagnostic and Interventional Radiology, Circolo Hospital, Insubria University, Varese, 21100 Italy; 8 Department of Medical Surgical Sciences and Advanced Technologies “GF Ingrassia”, UOSD I.P.T.R.A, University of Catania, University Hospital Policlinico “G. Rodolico-San Marco”, Catania, Italy

**Keywords:** Embolization, Radiology, Interventional, Angiography, Digital subtraction, Hematoma, Retroperitoneum

## Abstract

**Purpose:**

The purpose of this systematic review and meta-analysis was to evaluate the safety, technical, and clinical effectiveness of percutaneous Transarterial Embolization (TAE) in treating spontaneous retroperitoneal hematomas as well as assess treatment outcomes in patients who underwent target or empirical embolization.

**Materials and methods:**

Through the PubMed, Embase, and Google Scholar databases, an extensive search was performed in the fields of spontaneous retroperitoneal hematomas treated with transcatheter arterial embolization.

We collected pooled data on 141 patients from 6 separate articles selected according to the inclusion and exclusion criteria.

**Results:**

Technical success rate was 100% in all six studies, for both targeted and empirical embolization. The clinical success rate varied from 56.3 to 89.5%.

The total number of complications related to the embolization procedure was 10 events out of 116 procedures analyzed.

Empirical or empirical embolization was performed in three studies, where the source of active bleeding was not evident during DSA. A meta-analysis compared the rebleeding rates between targeted and empirical embolization groups. The odds ratio from pooled data from the three assessed studies (72 patients) showed no significant difference in rebleeding rates after empirical TAE compared with targeted TAE.

**Conclusions:**

TAE is a safe, effective, and potentially life-saving procedure for the treatment of life-threatening spontaneous retroperitoneal hematomas. Empirical and targeted TAE procedures demonstrate a relatively low risk of complications, compared to the high technical and relatively high clinical success rates.

## Introduction

Spontaneous Retroperitoneal Hematomas (SRHs) are defined as unprovoked bleeding into the retroperitoneum without any traumatic injuries or medical procedures [1]. The most frequent anatomical site for such bleeding episodes is the iliopsoas muscle, a composite of the psoas and iliacus muscles [1].

Available data suggest that psoas muscle hematomas are a relatively rare phenomenon, appearing in just 0.1–0.6% of individuals with predisposing factors but with relatively high mortality, up to 22% [1, 2].

SRHs frequently occur in elderly patients receiving therapeutic anticoagulation or antiplatelet therapy, often with multiple comorbidities. Other risk factors include cancer, hypertension, vasculitis, hematologic disorders, arteriosclerosis, or hemodialysis treatment [2]. The increased use of anticoagulants by the older population may be associated with the expected increase in the incidence of this pathology.

Spontaneous retroperitoneal hematomas have also been observed in patients hospitalized for COVID-19 [2], especially if on anticoagulation therapy, with an incidence 3–4 times higher than negative patients [3]. COVID-19 infection is associated with hyperactive fibrinolysis, a lack of coagulation factors, and overproduction of cytokines, which favor the formation of spontaneous hematomas [4, 5].

Accurate spontaneous retroperitoneal hematoma (SRH) diagnosis is crucial due to their often non-specific clinical presentation. Common symptoms, such as radiating back, flank, and hip pain, lumbar plexus neuropathy, and hemodynamic instability, can delay diagnosis and contribute to significant blood loss and high mortality rates [2]. Therefore, diagnostic imaging is indispensable in confirming the presence and characteristics of SRHs.

Computed Tomography (CT) is a crucial tool for effectively diagnosing SRHs. It offers rapid and precise localization of the hematoma, including its origin, extent, and associated signs. One such sign is the "hematocrit effect," characterized by a fluid–fluid level within the hematoma, reflecting the separation of denser cellular elements from the less dense serous fluid [6]. CT Angiography (CTA) further enhances diagnostic capabilities by detecting active bleeding within the hematoma, a well-defined area of increased attenuation within the arterial phase images, progressively expanding in subsequent phases. CTA's sensitivity is such that it can identify bleeding rates as low as 0.3 mL/min, a finding associated with treatment failure requiring a more aggressive approach [6].

Management of SRHs is still heavily debated but generally, in hemodynamically stable patients without any active bleeding, conservative treatment with fluid resuscitation, transfusions of packed RBCs, fresh frozen plasma, and cessation or reversal of anticoagulation is often the preferred approach. In case of failed conservative treatment with clinical or laboratoristic evidence of ongoing bleeding, digital subtraction angiography (DSA) followed by percutaneous transarterial embolization (TAE) is preferred nowadays to the classic open surgical approach.

Even without proof of active bleeding on DSA, an empiric embolization of the suspected bleeding vessel can be performed [7, 8].

Surgery might be reserved for patients in which the hematoma, with its mass effect, compresses neurovascular structures or in case of failed IR treatment [7, 9].

The purpose of this systematic review and meta-analysis was to evaluate the safety, technical and clinical effectiveness of percutaneous Transarterial Embolization (TAE) in treating spontaneous retroperitoneal hematomas as well as to compare treatment outcomes in the soubgroup of patients who underwent target or empirical approach.

Secondarily, evidence of active bleeding on Computed Tomography Angiography (CTA) and DSA, preferred embolization materials used, number of vessels embolized, complication rates, and mortality of this condition were assessed.

## Materials and methods

The review was conducted in accordance with the Cochrane Handbook of Systematic Reviews and Meta-analysis and the PRISMA statement guidelines. The study did not directly involve humans, and did not require the Institutional Review Board approval of our department.This systematic review was performed through the PubMed, Embase and Google Scholar databases, establishing the following keywords, medical subject headings (MeSH) and EMBASE Subject Headings (EMTREE): “embolization”,” spontaneous”, “retroperitoneal”, “hematoma”, “hemorrhage”, “bleeding”. The research was conducted using the following Boolean operators with the mentioned headings: (hematoma OR bleeding OR hemorrhage OR haemorrhage) AND (retroperitoneal) AND (spontaneous) AND (embolization OR embolisation). Titles, abstracts, and bibliography of publications were identified from the database results and screened according to inclusion and exclusion criteria. Subsequently, the full-text articles were examined to determine their eligibility.

### Study selection and data extraction

The starting search period was arbitrarily selected from 1991, and the screening lasted from September 2023 to October 2023, performed by two authors (S.T. and F.T.). The exclusion criteria were duplicate articles, case reports, case series with less than 10 patients, publications before the year 1991, editorials and commentaries, and articles not written in English.

Moreover, publications dealing with traumatic or non-spontaneous hematomas, treated exclusively with surgery or conservative treatment, or hematomas not originating from the retroperitoneum, or the posterior abdominal wall were also excluded.

Data was extracted from the publications, including the first author of the study, year of publication, type of study design, number of patients enrolled, patient characteristics (age, sex, hematoma location, hemodynamic state, and coagulation profile when available), and initial diagnostic approach (DSA or CTA).

Technical data regarding the embolization procedure were also extracted, such as embolizing material, embolized vessel, number of vessels embolized, target or empirical embolization, technical and clinical success rates, mortality, and complication rates.

### Outcome measure and statistical analysis

Technical success was defined by the proper delivery of the embolizing agent, confirmed by angiographic images of target vessel occlusion, without any evidence of bleeding as the procedure was finished. Clinical success indicates the absence of bleeding in subsequent imaging tests or laboratory results during the follow-up period. Collected data regarding technical and clinical success and complications were evaluated and compared.

Empirical embolization is defined as the embolization of a target vessel without angiographic proof of extravasation, typically guided by CTA findings in normal-appearing vessels [8, 10].

To standardize the report of complications described in the included articles, they were divided into major and minor categories according to the reporting standards of the Society of Interventional Radiology [11].

All data either explicitly declared by the authors of individual publications or inferred from the articles was included and analyzed. Unclear cases or data were marked as “Undeclared” or “Unspecified”.

Means and standard deviations represented continuous variables, while percentages represented categorical variables.

The Freeman-Tukey transformation was used to calculate the weighted summary proportions under the fixed and random effect model, and pooled proportions were evaluated with 95% CI.

All meta-analyses were conducted using random-effects models to better account for qualitative and quantitative heterogeneity between studies.

Comparative meta-analysis was performed using MedCalc software (MedCalc Statistical Software version 19.2., Ostend, Belgium). The results of the different studies and the respective and total odds ratios, with 95% CI, are illustrated in forest plots. *P* values < 0.05 were considered statistically significant. Categorical variables in retrospective studies were evaluated via odds ratio (OR) and were weighted using the Mantel-Haenszel method.

### Quality assessment and risk of bias

The authors assessed the six included studies using the Newcastle-Ottawa scale to assess the quality of nonrandomized studies.

## Results

This systematic review collected and identified 2573 articles to be screened and reviewed according to the inclusion and exclusion criteria. Successively, 494 duplicate articles were removed. After applying the exclusion criteria, a total of 1277 articles were identified. By assessing the titles and abstracts of these articles for appropriateness of the topic in question, 1255 articles were excluded. After applying the inclusion criteria, the total number was narrowed to 22 articles, including original research, reviews, and case series with more than eleven patients. The full-text versions of these twenty-two articles were examined, and ultimately, six publications were selected for the systematic analysis, published between 2015 and 2023 [2, 12–16]. As for the study design, all the included studies were retrospective in nature.

**Fig. 1:**
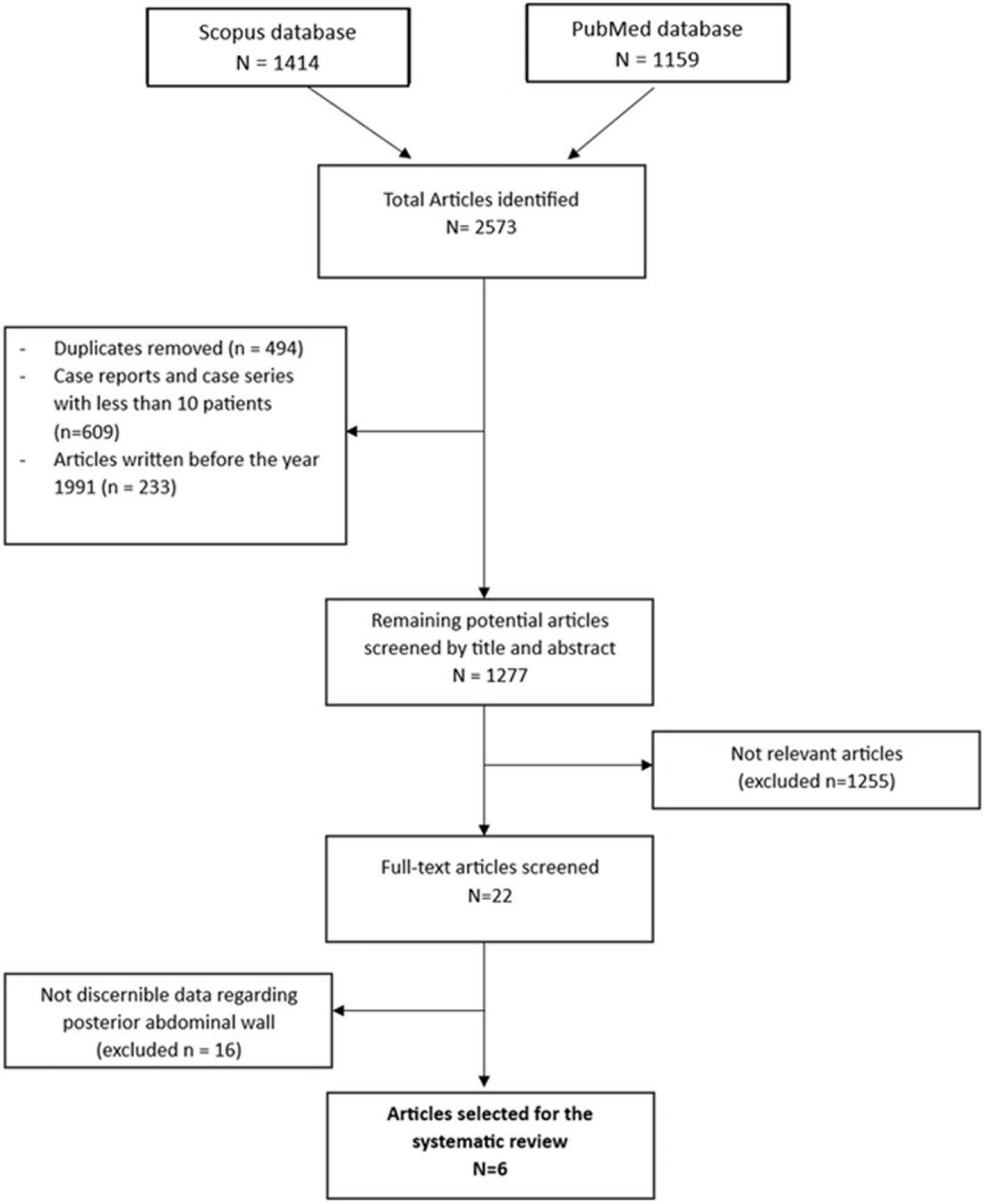
PRISMA flow diagram for study selection

A PRISMA flow diagram represented the study selection process (Fig. 1).

Based on the Newcastle-Ottawa scale for assessing the quality of nonrandomized studies, all six were deemed poor quality, mainly due to the lack of a control group or blinding of participants and outcomes (Table 1).

**Table 1:** Newcastle-Ottawa scale for assessing the quality of nonrandomized studies

Newcastle Ottawa Scale		Author					
		Dohan et al. [12]	Klausenitz et al. [13]	Lukies et al. [14]	Tani et al. [15]	Tiralongo et al. [2]	Wang et al. [16]
Selection	Representativeness of exposed cohort	-	-	-	-	-	-
	Selection of nonexposed cohort	-	-	-	-	-	-
	Ascertainment of exposure	*	*	*	*	*	*
	Outcome not present at baseline	-	-	-	-	-	-
Comparability of cohorts		-	-	-	-	-	-
Outcome	Assessment of outcome	*	*	*	*	*	*
	Sufficient follow-up duration	*	*	*	*	-	*
	Adequate follow-up	-	*	*	-	-	*
Total score/ Quality		3/poor	4/poor	4/poor	3/poor	2/poor	4/poor

Good quality is defined as 3 or 4 (*) in the selection domain AND 1 or 2 stars in comparability domain AND 2 or 3 stars in outcome domain. Fair quality is defined as 2 stars in the selection domain AND 1 or 2 stars in the comparability domain AND 2 or 3 stars in the outcome/exposure domain. Poor quality is defined as 0 or 1 star in the selection domain OR 0 stars in the comparability domain OR 0 or 1 stars in the outcome/exposure domain

### Demographic characteristics

Data from a total of 141 patients, as reported in these 6 articles, was pooled and analyzed. As for the characteristics of the study population, 80 patients were males and 61 were females.

Meta-analysis of pooled proportions showed an overall 56.7% of males (95% CI, 21.29–86.12; I2 66.95%). The mean age across the included studies is 67.4 ± 14 years.

Demographic data on gender, age and pre-procedural hemoglobin level can be viewed in Table 2.

**Table 2:** Demographic characteristics of the study population

First author, year	Number of patients	Male	Female	Mean Age	Mean preprocedural Hb (g/dL)
Dohan et al., 2015 [12]	36	12	24	74 ± 14.2	7.4 ± 2.0
Klausenitz et al., 2020 [13]	30	17	13	71.9 ± 9.8	7.11 ± 1.61
Lukies et al., 2023 [14]	16	13	3	64.5 ± 21.5	8.45 ± 1.53
Tani et al., 2019 [15]	19	10	9	69.6 ± 10.7	7.9 ± 2.0
Tiralongo et al., 2022 [2]	24	17	7	72.7 ± 11.2	7.96 ± 1.35
Wang et al., 2016 [16]	16	11	5	51.5 ± 16.4	7.41 ± 2.14

In all studies, except for Tiralongo et al. [2], anticoagulation status was noted and reported, due to the causative link between anticoagulation and spontaneous retroperitoneal hematomas (87.6% of all patients included were on anticoagulation therapy).

Regarding hemodynamic instability, all available data were extracted and pooled, except from Tiralongo et al. [2] where these data were not specified. 87/102 patients (85,3%) with SRHs were hemodynamically unstable.

Table 3 summarizes the data concerning anticoagulation status and hemodynamic instability deriving from each study. Only the population with SRHs was extracted from Dohan et al. (21 out of 36 patients) [12].

**Table 3:** Summary of anticoagulation status, hemodynamic instability, CTA and DSA evidence of active bleeding, embolized vessels, number of vessels, embolic materials, technical, clinical, rebleeding, complication rates and type of complications

First author, year	Number of Patients	Anticoagulation therapy	Hemodynamic instability	CTA evidence of active bleeding	DSA evidence of active bleeding	Embolized vessels^a^	Number of embolized vessels	Embolic Material^a^	Technical Success rate (%)	Clinical Success rate (%)	Rebleeding Rate (%)	Complications rate (%)	Complications (number and type)
Dohan et al., 2015 [12]	36 (21 with SRH)	21 (100%)	21 (100%)	Undeclared	Undeclared	LA (33), ILA (11)	44	Unspecified	100%	Undeclared	5 (23.8%)	4.7%	1 minor complication (infected retroperitoneal hematoma)
Klausenitz et al., 2020 [13]	30 (29 embolized)	30 (100%)	30 (100%)	28 (93.3%)	22 (73.3%)	LA (22), ILA (7), DCIA (4), SGA (1), SLA (1)	43	NBCA (15); Coils (10); NBCA + Coils (4)	100%	65.5%	7 (24%)	6.9%	2 minor complications (access site hematomas)
Lukies et al., 2023 [14]	16	11 (69%)	6 (38%)	13 (81,2%)	Undeclared	Undeclared	Undeclared	Gelfoam (7); Coils (9); NBCA (1)	100%	56.3%	5 (31.2%)	0%	No complication reported
Tani et al., 2019 [15]	19	18 (94,7%)	14 (74%)	16 (84,2%)	15 (78.9%)	LA (14); ILA (11); DCIA (9); SGA (3); ICLA (3); IEA (5); SLA (1); IIA (1); Anastomosis (1)	48	Gelfoam (11); Gelfoam + NBCA (3); Gelfoam + Coils (4); Coils (1)	100%	89.5%	2 (10.5%)	5.2%	1 minor complication (transient neuropathy of right upper thigh)
Tiralongo et al., 2022 [2]	24	Undeclared	Undeclared	20 (83%)	20 (83%)	LA (19); ILA (17); DCIA (5); ICLA (2); IEA	69	Gelfoam (11); Gelfoam + Coils (13)	100%	71%	7 (29.2%)	4.1%	1 minor complication (small dissection of deep circumflex artery)
Wang et al., 2016 [16]	16	4 (25%)	16 (100%)	9 (56%)	7 (88%)	LA (5); ILA (1); IIA (1); Suprarenal Artery (1)	Undeclared	Coils (4); Coils + Gelfoam (3)	100%	85.7%	1 (14.3%)	0%	No complication reported

*LA* Lumbar artery, *ILA* Iliolumbar artery, *DCIA* Deep circumflex iliac artery, *SGA* Superior gluteal artery, *SLA* Sacral lateral artery, *ICLA* Intercostal artery, *IEA* Inferior epigastric artery, *IIA *Internal iliac artery

^a^In brackets the number of patients

### Pre-procedural findings

Almost all patients with SRHs, as documented within the six studies reviewed, underwent CT to locate the bleeding source, 123/141 patients (87,2%) in total. CTA showed active bleeding in 86/105 patients, with the meta-analysis of pooled proportion, which demonstrated an overall 81.6% active bleeding detection rate (95% CI, 0–82.80; I2 53.26%).

As noted earlier, concerning Dohan et al. [12], 21 out of the total 36 patients had a retroperitoneal hematoma, whereas all the other patients included in the remaining five studies suffered from SRHs. Dohan et al. [12] did not specify the exact number of patients with active bleeding sites from the retroperitoneum, therefore it was deemed undeclared in this review.

As for DSA evidence of active bleeding, data from Dohan et al. [12] and Lukies et al. [14] could not be extracted, as they were not specified. In the four remaining publications, DSA showed evidence of active bleeding in 64/81 patients who underwent angiography. Pooled data from the six studies showed a weighted proportion of 77.9% (95%CI, 0–58.66, I2 0%) of active bleeding sites identified on DSA. CTA and DSA evidence of contrast extravasation deriving from individual studies are summarized and compared in Table 3.

### Procedural findings and outcome

Transarterial Embolization (TAE) was performed in 116/141 patients (82,3%) across all six studies, either targeted or empirical. As for the embolized vessels, data was collected from all studies except from Lukies et al. [14], which was unspecified.

Data regarding the number of embolized vessels was individually collected from four studies: Dohan et al. (*n* = 44), Klausenitz et al. (*n* = 43), Tani et al. (*n* = 48), and Tiralongo et al. (*n* = 69) [2, 12, 13, 15]. The total number of embolized vessels for the treatment of SRHs was 204. In the remaining two studies [14, 16], this information was not available. The most frequently embolized vessels were: Lumbar arteries (*n* = 93; 45.5%), Iliolumbar arteries (*n* = 47; 23%), Deep Circumflex Iliac (*n* = 18; 8.8%), Inferior Epigastric (*n* = 6; 0.29%), Intercostal (*n* = 5; 0.25%), Superior Gluteal Arteries (*n* = 4; 0.2%) and other arteries in single instances (tot *n* = 31; 15.2%). Individual data regarding the embolized vessels, their number, and embolic material was summarized in Table 3.

As for the embolic materials utilized, the most frequently deployed were: Gelfoam (*n* = 29; 30.2%); Coils (*n* = 24; 25%); Gelfoam + Coils (*n* = 20; 20.8%); N-butyl cyanoacrylate (NBCA) (*n* = 16; 16.7%); NBCA + Coils (*n* = 4; 4.2%); NBCA + Gelfoam (*n* = 3; 3.1%). Data regarding Dohan et al. [13] could not be extracted because unspecified. Lukies et al. [14] mentioned utilizing a combination of Gelfoam with coils or NBCA in an unspecified number of patients excluding the aforementioned ones, and as a result, it was not included in Table 3.

Technical success rate was 100% in all six studies, for both targeted and empirical embolization. Although not consistent, data regarding clinical success were extracted from each publication. The clinical success rate was collected from all studies except from Dohan et al. [12], and varied from 56.3 to 89.5%. According to the objective of this study, the definition of clinical success indicates the absence of signs of bleeding in subsequent imaging tests or laboratory results during the follow-up period. Considering this definition, a rebleeding rate could be retrieved across the studies. Meta-analysis of pooled proportion of across the studies showed a total rebleeding rate of 24.17% (95%CI, 0–59.90, I2 0.0%). Technical success, clinical success, and rebleeding rates were collected and compared in Table 3.

The total number of complications related to the embolization procedure was 5 events out of 116 procedures analyzed. Meta-analysis of pooled proportion showed an overall complications rate of 5.764% (95%CI, 2.354 − 11.498, I2 0%). No major embolization-related complications were noted across the included articles except for Lukies et al. [14], where out of the 16 treated patients, four died for ongoing instability after treatment, and two others died during surgical hemostasis and for bacteremia within two weeks after the embolization. A summary of the number, rate, and specific types of complications reported in each study is shown in Table 3.

The mortality rate at 30 days ranged from 5.2 to 43.4% (mean 24.3%) in three of the six included studies [13–15]. The remaining three studies did not specify this information. Data regarding survival rates were unavailable in the included studies and, therefore, were not collected.

Empirical embolization was performed in three studies [2, 13, 15] where the source of active bleeding was not evident during DSA, whereas in two studies [14, 16] it was not conducted. Dohan et al. did not specify the number of empirical procedures conducted specifically for spontaneous retroperitoneal hematomas [12]. Technical success was 100% for all the empirical embolization procedures performed. Data regarding the number, rate, and technical success of empirical embolization is summarized in Table 4.

**Table 4:** Number, rate and technical success of blind embolization procedures

First author, year	Number of empirical procedures	Technical success (%)
Dohan et al., 2015 [12]	Unspecified	100%
Klausenitz et al., 2020 [13]	6 (20.7%)	100%
Lukies et al., 2023 [14]	0	Not blind
Tani et al., 2019 [15]	4 (21%)	100%
Tiralongo et al., 2022 [2]	4 (17%)	100%
Wang et al., 2016 [16]	0	Not blind

### Subgroup Analysis

**Fig. 2:**
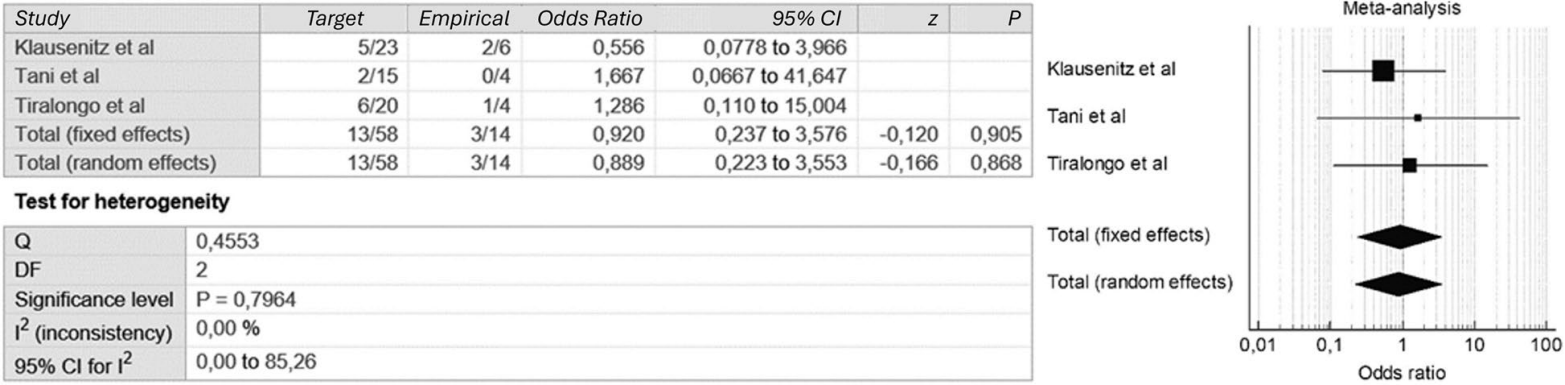
Comparative meta-analysis and forest-plot for comparing rebleeding rates after targeted (“Target”) and empirical embolization (“Empirical”) of the included studies

A meta-analysis using the Mantel – Haenszel method was conducted to pool and correlate the results from three different studies where empirical embolization was performed [2, 13, 15] (Table 5). Odds ratio (OR) with a corresponding 95 % confidence interval (95 % CI) was used to compare the rebleeding rates between targeted and empirical embolization groups. The Cochrane chi-squared (Q) test was utilized to determine if the observed differences in outcomes were due to chance alone. If the calculated *P*-value was less than 0.05, it indicated a statistically significant relationship between the two groups. Statistical heterogeneity across studies was evaluated using I2, with a value greater than 50 % indicating substantial heterogeneity. The odds ratio from pooled data from the three assessed studies (total of 72 patients) showed that there was no significant difference in rebleeding rates after empirical TAE compared with targeted TAE (OR 0.92, 95 %CI 0.23–3.57). No rebleeding episodes were noted after empirical TAE in the study by Tani et al. [9]. No statistically significant heterogeneity was found among the studies (*P* = 0.796, I2   0.0 %, 95%CI  0–85.26). The aforementioned results are illustrated and displayed in the forest plot in Fig. 2.

**Table 5:** Target vs. Empirical embolization groups with rebleeding rates

First author, year	Total patients number	Targeted Embolization (TE) number	Empirical Embolization (EE) number	TE Rebleeding	EE Rebleeding
Klausenitz et al. [13]	29	23	6	5 (21.7%)	2 (33.3%)
Tani et al. [15]	19	15	4	2 (13.3%)	0 (0%)
Tiralongo et al. [2]	24	20	4	6 (30%)	1 (25%)

## Discussion

The role of TAE in the management of spontaneous retroperitoneal hematoma has been investigated in many studies, representing a safe and effective alternative to surgical treatment in hemodynamically unstable patients or patients unresponsive to conservative treatment [13, 14]. However, there are not yet valid recommendations on using TAE in hemodynamically stable patients, especially since the cessation or reversal of anticoagulation can frequently resolve this condition. As reported by Warren et al. and Lukies et al., the conservative treatment of SRHs may successfully manage even shocked or hemodynamically unstable patients [14, 17].

This review corroborated the association between anticoagulation therapy and SRHs [12] a mean of 87.6% of the included patients were anticoagulated (Table 3) as confirmed by previous reports [17].

SRH is typically a condition of the old population, due to the anticoagulation therapy and the different comorbidities, with a mean age of 67.4 ± 14 years across the study population, ranging from 51.5 ± 16.4 years to 74 ± 14.2 years [12, 16].

Comorbidities such as coagulopathies (hemophilia A, Factor X deficiencies), or coexisting chronic renal failure can favor an earlier presentation of SRHs [16].

Regarding the diagnostic framework, CT plays a fundamental role in identifying and characterizing the location, volume, and presence of active bleeding, especially since clinical symptoms are often vague, with a high sensitivity (86.7–93.3%) [13, 15].

CT can detect the extent of the hematoma into the retroperitoneal space, an important finding related to higher mortality and rebleeding even after interventional treatment [2, 19].

Across the population included in the six studies reviewed, 87.2% of patients underwent preprocedural CTA, which identified the presence of active bleeding in 81.6% of cases (range 56.2 − 93.3%).

According to Tani et al. [15], the specificity of CT was fairly low (50%), therefore the authors suggest contrast enhanced CT (CE-CT) as a screening investigation to guide the decision for angiography.

Dohan et al. suggest that factors like anticoagulation therapy, intermittent bleeding due to spasm or hypotension, and soft tissue tamponade in elderly patients with atherosclerosis can contribute to negative findings on CE-CT [12].

For this reason, the absence of signs of active bleeding at CE-CT does not exclude the need for angiography and subsequent TAE [15].

In four out of the six studies, data regarding DSA evidence of active bleeding was extracted, demonstrating that 77.9% of patients who underwent DSA showed signs of contrast extravasation (range 73.3 − 88%).

Subsequent TAE was performed in 82.3% of all patients (116/141). In those patients with no CTA evidence of contrast extravasation and absent or indirect signs of bleeding during DSA (vessel irregularities, vasospasm), empirical embolization was conducted. In particular, in three of the included studies [2, 13, 15], a total of 14 out of 72 patients received empirical treatment (19.4%).

The meta-analysis conducted to compare the rebleeding rates between targeted and empirical embolization groups did not reveal any statistically significant differences or heterogeneity (*P* = 0.796; I2 0.0%). Recent studies confirm this finding, emphasizing that the efficacy and safety of empirical embolization are comparable to targeted embolization in the treatment of spontaneous abdominal wall hematomas [18, 19].

However, there aren’t yet definitive indications in the use of this approach, and therefore the decision should be guided by clinical-radiological findings and the possibility of promptly managing any complications or IR treatment failure through surgical intervention [2, 9].

The technical success rate of TAE was 100% across all the articles reviewed, including targeted and empirical embolization, proving that TAE is a safe and effective option in managing SRHs.

Considering the six included studies, the most frequently embolized vessels were the lumbar and iliolumbar arteries, respectively (68.5% of all bleeding vessels). This data was in line with previous publications and it was explained by the typical location of SRHs in the iliopsoas muscle [13].

Regarding the embolic material, the present review showed that the most frequently utilized was Gelfoam (30.2%), followed by Coils (25%), NBCA (16.7%), and various combinations thereof.

It is worth noting that except for Klausenitz et al. [13] where it was not employed, Gelfoam was utilized in all the remaining studies, either alone or in association with coils or NBCA.

As reported by Tani et al. [15] the underlying coagulopathy may favor the use of a temporary agent like Gelfoam in conjunction with the reversal or cessation of anticoagulation treatment, even though it carries the risk of recanalization if the coagulation status is not properly corrected.

The study by Klausenitz et al. was the only one where NBCA was the most frequently utilized agent (65.5% of patients) either alone or with coils [13].

As reported by Takasawa et al., NBCA is a highly effective embolic agent, because it achieves total occlusion of the bleeding vessels even in individuals with disrupted coagulation parameters [20]. Furthermore, it allows greater control of the extent of embolization within the target vessel, reducing the risk of rebleeding when compared to temporary agents [20].

The clinical success ranged from 56.3 to 89.5% among the five studies reviewed. Data regarding clinical success was inherently nonhomogeneous because of the different definitions across the six studies of “clinical success”, and the differing lengths of the follow-up period.

As previously stated in the present review, clinical success is defined as the absence of signs of bleeding in subsequent imaging tests or laboratory results during the follow-up period. Coherently with the included publications, this review confirmed that rebleeding is a common occurrence after embolization procedures in patients with SRHs, showing a rebleeding rate of 24.17% across the six studies reviewed (ranging from 10.5 to 31.2%).

This may be because coagulation parameters were not corrected rapidly enough, or because of the stretching and secondary rupture of vessels caused by the expanding hematoma or its tamponade effect [21].

Furthermore, the rich collaterals network created by the lumbar arteries in the retroperitoneum can facilitate, in conjunction with predisposing conditions (arteriosclerosis, coagulopathies, vasculitis), the development of multiple foci of vessel disruption and recurrent bleeding, sometimes not detected during the first DSA [13].

In case of ongoing bleeding or hemodynamic instability after the first TAE, the procedure can be repeated until the vessel responsible for the extravasation is located and occluded, and/or until stabilization of the hemodynamic condition in cases of empirical embolization [2, 7, 13].

Technical success for re-embolization is still high, and any subsequent clinical failures are not necessarily related to the embolization technique [12, 14, 15].

This review showed a low complication rate of 5.76% across all studies, ranging from 0 to 6.9% (5 events out of 116 procedures). In particular, according to the SIR reporting standard, only 5 minor complications were noted, ranging from access site hematomas to a small iatrogenic arterial dissection. This review showed that the 30-day post-treatment mortality rate was 24.3% (ranging from 5.2 to 43.4%) in line with the reported rates in previous publications [1, 7, 21].

The main limitations of this systematic review are the retrospective nature of the included cohort studies, the majority of which are single-arm retrospective series only, without a cohort comparison arm (e.g. patients treated with conservative management), which inevitably introduces selection bias and limits scientific evidence. Furthermore, the relatively low number of studies and the subsequent scarcity of the included population reduce the validity of the present review. However, considering that SRHs are potentially life-threatening emergencies these limits were expected since the planning of prospective studies or clinical trials is much more complex. Another limitation of this review is the heterogeneity of data regarding the follow-up period across the included articles, although this review aimed to assess the safety and efficacy of TAE in managing this emergency condition.

## Conclusions

TAE is a safe, effective, and potentially life-saving procedure for the treatment of life-threatening spontaneous retroperitoneal hematomas. Empirical and targeted TAE procedures demonstrate a relatively low risk of complications, in contrast to the high technical and relatively high clinical success rates, as already shown in other publications. The meta-analysis revealed no statistically significant difference in rebleeding rates between targeted and empirical embolization. Additional prospective studies comparing conservative and endovascular management are also necessary to provide insight into this potentially life-threatening condition.

## Data Availability

Not applicable.
